# Impact of pharmacist-led medication management in care transitions

**DOI:** 10.1186/s12913-017-2684-3

**Published:** 2017-11-13

**Authors:** Seungwon Yang

**Affiliations:** Department of Practice and Pharmacy, UCL School of Pharmacy, London, UK

**Keywords:** Post-discharge medicines management, Pharmacist intervention, Care transitions, Pharmacist intervention, Telephone follow-up, Hospital readmission rates

## Abstract

**Background:**

When patients are discharged from hospital to home, it is a highlighted vulnerable period for which medication - related problems are prevalent. Researchers have proposed a telephone follow-up intervention as a means to reduce hospital readmissions. However, the outcome of the intervention with the engagement of pharmacists in managing patients’ medicines after discharge has not been well explored. The objectives of this study were (1) to determine whether a pharmacist telephone follow-up intervention focusing on patients’ medicines management support is associated with a reduction in 30-day readmission rates and (2) to describe the number and types of pharmacist interventions in care transitions.

**Methods:**

This was a case-cohort study conducted in two acute hospitals in the UK. Pharmacists performed a telephone follow-up intervention to discharged patients to provide medicines management support. Patients who received pharmacist telephone follow-up calls within 14 days of discharge formed the intervention group. A subset of medical patient population discharged in the month of May 2013 formed the comparison group. During a series of two-telephone follow-up, pharmacists identified post-discharge pharmaceutical problems and provided patient-tailored interventions accordingly. The impact of pharmacist interventions was assessed using a risk assessment matrix tool by two senior pharmacists. Overall 30-day readmission rates in the intervention group were measured and compared with the comparison group using a chi-square test.

**Results:**

Between 5^th^ and 25^th^ June 2013, a total of 62 medical patients participated in the study. Pharmacists provided 192 interventions as a result of pharmacist telephone follow-up intervention. The most prevalent type of interventions was the provision of drug information (*n*=40), followed by screening patient adherence (*n*=30) and advising on adverse drug reactions (*n*=27). The impact of interventions was assessed, and 49.3% of the identified risks intervened by pharmacists were associated with moderate risk. The 30-day readmission rates in the intervention group were 11.3% compared to 9.0% in the control group (*p* = 0.376); this was not statistically significant.

**Conclusions:**

A pharmacist TFU intervention did not show a benefit in 30-day hospital readmissions. However, a pharmacist TFU intervention was an effective method to solve or avoid critical pharmaceutical problems. A future study using a larger scale trial is warranted.

## Background

Managing patients’ medicines after hospitalisation plays an integral part in an efficient discharge process to ensure patients’ safety and optimise health outcomes [1, 2]. It is a highlighted vulnerable period for discharged patients to continue the consistent degree of care and manage their medicines, often accompanied by poor communication with healthcare providers, poor understanding of prescribed medications, poor adherence, or inadequate monitoring of adverse effects [3, 4].

A substantial percentage of patients encounters medicine-related problems shortly after hospital discharge [5]. Patients discharged from a hospital to a home setting are related to an increased risk of critical medication discrepancies as the alteration of medicines commonly occurs in care transitions [6]. Recent studies have demonstrated the significance of medicine-related problems in care transitions [6–8]. One in five hospitalisations was attributed to post-discharge adverse events within 30 days post-discharge, of which 72% of these adverse events were responsible for drug-related problems [9]. The costs of unplanned hospital readmissions have been a major concern in the U.K. The National Health Service (NHS) estimated that approximately 3~11% of patients were readmitted to the hospital within four weeks post-discharge [10]. Readmissions account for a significant amount of hospital expenditure, costing the NHS an estimated 1.6 billion pounds in 2010 [8]. Therefore, avoiding rehospitalisation has become a priority for policy makers in the U.K. The government has announced that hospitals should not be reimbursed if patients are readmitted within 30-day of discharge and should only receive a “30% marginal rate for emergency department visits above their 2008/2009 baseline” [11]. *Billings* et al. *(2012)* suggested that the “30-day readmission risk” could be set as an endpoint target for healthcare providers to organise discharge planning efforts and post-discharge care support [12]. With the aim of curbing hospital expenditure through reducing 30-day readmission rates, one of the NHS schemes encourages healthcare providers to plan and maintain a provision of care support for the first 14 days of discharge [11]. The 14-day monitoring has been emphasised because “the first 2–3 weeks” post-discharge was regarded as a crucial window period for the prevention of subsequent admissions [13].

The implementation of post-discharge interventions has emerged as a critical strategy to provide optimal health outcomes to patients in care transitions [14]. With strategies to provide cost-effective interventions, a telephone follow-up (TFU) has been proposed by researchers as a relatively uncomplicated and plausible tool to maintain patient care without interrupting communication between healthcare providers and patients across continuity of care [15].

Current evidence evaluating the benefit of a TFU intervention were inconsistent and mixed. Only a few studies have shown a reduction in 30-day readmission rates with statistical significance following the TFU. Although an expanding role of pharmacists has been emerged in assisting the post-discharge process, the outcomes of pharmacist interventions linked to readmissions rates have not been well-defined. No researcher has addressed the extents to which the engagement of pharmacists focusing on managing patient’s medicines delivered through telephone follow-up can have an impact on the rates of hospital readmissions, particularly based in the U.K.

A pharmacist post-discharge TFU service was piloted in medical patients. This new pharmacy service involved pharmacists contacting patients discharged to home via telephone in the immediate post-discharge period to provide medicines management support. The objectives of this study were to describe the number and types of pharmacist intervention in medical patients after hospital discharge and determine whether a pharmacist telephone follow-up intervention focusing on patients’ medicines management support is associated with a reduction in 30-day readmission rates.

## Methods

### Study design and setting

This was study a case-cohort conducted at two acute hospitals (part of NHS Trust), located in Buckinghamshire in the UK. Patients who received the intervention formed a prospective cohort group. The intervention group was medical patients who were discharged from the emergency room or medical general wards and received pharmacist telephone follow-up calls within 14 days of discharge between 5th and 25th June 2013. A comparison cohort group (control group) was a subset of patient populations who were discharged from the emergency room or general medical wards in the month of May 2013. The medical patients were targeted for the TFU intervention because they tend to take multiple medications as well as have high readmission rates within the Trust. Before the start of data collection, the pilot study was conducted on three patients, but no significant changes were made.

This study was approved by the Human Research Ethics Committee at UCL School of Pharmacy. A verbal informed consent was obtained from all participants prior to enrolment. As this study was neither intrusive nor risky to participants and researcher and was observational in nature, the ethics committee waived the need for written informed consent form from the participants. Participation in this study was voluntary, and confidentiality was maintained throughout the study.

### Participant inclusion and exclusion criteria for the intervention group

The study population consisted of home-dwelling adult patients aged 18 or older and discharged from emergency medicines or general medical wards. The sample size was calculated to estimate the number of subjects needed to detect meaningful effects of a reduction in hospital readmission rates. The rate of rehospitalisation for the TFU group was expected to have a 50% reduction in readmissions from 11% (NHS Institute for Innovation and Improvement, 2005–2013) to 5.5% [16]. Thus, a total sample size of 416 gives a power of 0.80 and a significant level of alpha = 0.05. Further readmission and emergency visit were counted only when patients were readmitted to one of two hospitals. Patients in the intervention group were excluded if they were transferred to the site other than homes, such as nursing home or other acute hospitals. Patients must have access to a working phone and be an English-speaker or live with a person who can speak English to communicate with pharmacists.

### Description of a telephone follow-up intervention (TFU)

At the point of discharge, pharmacists offered eligible patients a TFU service with a verbal description. All consented patients were referred to the structured pharmacist TFU service in addition to usual care. A telephonic discharge follow-up was performed by senior pharmacists daily from Monday to Friday during their working shift. Pharmacists performed post-discharge TFU calls on two occasions; at 2–7 days and after 10-days post-discharge. To ensure the coherency of TFU interview among the pharmacists, participating pharmacists underwent the training about consultation skills and a formatted script was provided. At the start of the telephone interview, patients were asked to bring all current medicines to the telephone. The telephoning pharmacists reviewed the list of patients’ medicines from hospital discharge summary and current self-reported medicines taken after discharge. During the TFU, pharmacists asked patient questions in line with a formatted script and identified existing pharmaceutical problems. Then, telephoning pharmacist made interventions to solve or ameliorate the existing/potential problems. Each completed or attempted TFU including the details of the interview was documented on a pharmacist interview form. The pharmacist reviewed pharmaceutical problems if further action was required and ensured that appropriate measures had been taken.

### Key content of the TFU

A pharmacist TFU intervention was focused on optimising medicines management through recognising and solving any pharmaceutical problems as well as answering patients’ questions concerning their post-discharge medicines. The TFU interview was tailored to individual patients’ needs.

1. During the first call, pharmacists

- performed in-depth medicines use review; pharmacists ensured that patients fully understood the discharge medicines and appropriate medicine use
- addressed any pharmaceutical problems, any side effects, adherence issues, and inadequate supply of medicines for next 2 weeks
- solved problems and answered any questions/concern/issues from patients regarding their medicines.

2) During the second call, pharmacists

- questioned about any changes concerning medicines since the first call
- followed up any issue(s) identified during the first phone call
- ascertained whether patients had enough supply for the next two weeks after discharge

### Risk assessment

The impact of pharmacist interventions was assessed by two highly qualified pharmacists (qualification with Band 9) according to criteria using the National Patient Safety Agency (NPSA) risk matrix [17]. The senior pharmacists identified the risk of the pharmaceutical problems, which could have potentially rendered harms to patients. Two senior pharmacists independently scored the identified risks and graded to yield the severity level of the risk. Any discrepancies were solved by consensus. The assessment of identified risk was categorised into four steps: (1) actual harm to patient, (2) harm if interventions undetected, (3) likelihood of recurrence, and (4) Scoring and grading risk. An association between patient characteristics and the grade of risk was assessed.

### Data collection

For the intervention group, individual patients were assigned with own identity code to protect patients’ confidentiality throughout the study. Patient discharge date and discharge diagnosis were obtained from hospital discharge summaries. Demographic data, such as patient age and a number of medications taken, and dependent data on the types and number of pharmacist interventions made were prospectively gathered from pharmacist action/interview sheets. Data on risk assessment were also prospectively obtained from risk/intervention evaluation sheets. Additionally, for the intervention group, independent data on 30-day hospital readmission was obtained retrospectively through manual review of electronic medical records, the “Patient Management System” after a period of 30-days post-discharge. For the comparison cohort group, independent data on 30-days readmission rates were obtained from the information department of the NHS Trust. However, it was not possible to obtain individual baseline data for the comparison cohort because hospital policy restricted an access to patient information.

### Outcomes measures

1. The primary outcome was to determine whether a pharmacist TFU intervention focusing on medicines management support has an impact on 30-day all-cause readmission rates in medical patients after hospital discharge.
2. The secondary outcome was the number and types of pharmacist interventions made as a result of post-discharge TFU intervention.

### Data analysis

Data processing and data analysis were conducted using the Statistical Programmed for Social Science (SPSS) version 19. Discharge date and readmission date were used to determine 30-day hospital readmissions. To compare the rates of readmissions, historical data was assigned to the control cohort group. 30-day hospital readmission in the intervention group was calculated and compared with the control group to detect a significant difference. The comparison of hospital readmission rates between the intervention and the control groups was evaluated using a chi-square test. The differences in the mean duration of telephone calls between the first and the second calls were analysed using a paired t-test. *P* < 0.05 was defined as statistical significance. Missing data were excluded from the data analysis. Additional descriptive statistics, such as mean and frequency, were used to describe other study outcomes.

## Results

A total of 62 medical patients were recruited into the study. These patients received a series of two TUF and formed the intervention group. The mean age of the patient was 63 ± 19.1 years, with the average of 2.1 ± 1.4 medical conditions and 6.7 ± 3.4 medicines taken per patient (Table 1).

**Table 1:** Baseline characteristics of patients in the intervention group

Variable	*n* (%)
Age	
Mean age, y (SD)	63.3 (19.1)
Age range, y	19–92
≤ 40 years, y	8 (13.3)
41–60 years, y	16 (26.7)
61–80 years, y	26 (43.3)
≥ 81 years, y	10 (16.7)
Medical conditions (n)	
Mean number of medical conditions, n (SD)	2.1 (± 1.4)
Range, n	1–8
Number of medicines taken per patient	
Mean number of medicines taken per patient, n (SD)	6.7 (± 3.4)
Range	2–18
≤ 4	19 (30.6)
5–8	25 (40.3)
9–11	13 (21.1)
12–15	3 (4.8)
≥ 16	2 (3.2)
Total number of patients	62 (100)

*Abbreviations*: *n* number, *SD* standard deviation, *y* years

### Time commitment involved

The mean duration of the first call vs. the second call was 14.46 ± 11.4 min and 5.87 ± 5.6 min per patient, respectively (*p* < 0.0001). The average length of time for both calls was 10.17 (±4.3) minutes (Table 2).

**Table 2:** The Duration of TFU (*N* = 62)

Duration of telephone calls	Time (min.)
Mean duration of Call# 1	14.46 (± 11.4)
Call# 1 range	1~45
Mean duration of Call #2	5.87 (± 5.6)
Call #2 range	1~25
Mean duration of call #1 and #2^*^	10.17 (± 4.3)

*Abbreviations*: *TFU* telephone-follow-up, *N* number, *min* minutes

^*^ *p*-value <0.0001 using a paired t-test

### Type and number of pharmacist interventions

A total of 192 pharmacist interventions were made as a result of a pharmacist TFU, equating to the average of 3.56 interventions per patient. Many different types of pharmacist interventions were made. The most frequent intervention was patient counselling, provided to 74% of patients, and followed by advising on adherence issues (in 54% of patients), reviewing adverse drug reaction(50.0%), instruction on the duration of therapy (46.3%) (Table 3).

**Table 3:** Type and number of pharmacist interventions

Type of Pharmacist Intervention	Number of Pharmacist Interventions	% of pharmacist interventions per patient population
Clarifying medication regimen	13	24.1%
Reviewing indication	16	29.6%
Reviewing dosage/direction	15	27.8%
Instruction on duration of therapy	25	46.3%
Reviewing adverse drug reaction	27	50.0%
Instruction on Adherence/Screening for barriers to adherence	30	55.6%
Reviewing drug/food interaction	3	5.6%
Providing drug information	40	74.1%
Ensuring the sufficient supply of medication	19	35.2%
Others	5	9.3%
Total	192	100%

Others: checked follow-up with physician, recommended INR level monitor or advised to discuss

### Risk assessment

Based on the pharmaceutical problems intervened by pharmacists, 75 incidents (identified risk) which could have potentially rendered harm to patients were identified from two senior pharmacists. Identified risks were scored and assigned grades to identify the severity level at which the risks were solved or managed by pharmacist interventions. The risk grade was stratified by four different levels. Almost half (49.3%) the risks were graded as “moderate” and 9.3% were “Extreme risk” (Table 4).

**Table 4:** Risk Assessment using the National Patient Safety Agency (NPSA) risk matrix (*N* = 75)

Level of risk	Frequency of risk, N (%)
Extreme Risk	7 (9.3)
High Risk	22 (29.3)
Moderate Risk	37 (49.4)
Low Risk	9 (12.0)
Total	75 (100)

*Abbreviation*: *N* number

### Hospital readmission rates

**Fig. 1:**
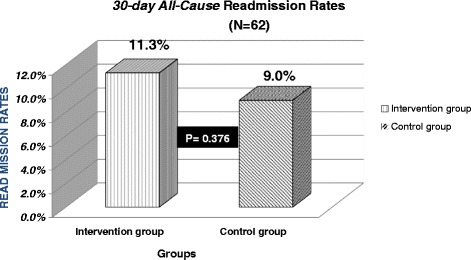
30-day all cause readmission rates

Of 62 patients in the intervention group, seven patients (11.3%) were readmitted to hospital in the first 30-days post-discharge. For the control group, a total of 1908 patients were discharged from the medicine directorate in May 2013 and were therefore selected for the control group. 172 of which were readmitted to the hospital within 30 days of discharge, accounting for 9% of Readmission rates. Therefore, the 30-day readmission rate was 11.3% in the intervention group compared to 9% in the control group (*p* = 0.376) (Fig. 1.).

## Discussion

There has been a strong drive to reduce hospital readmissions. Its avoidance can be a crucial marker for ensuring the quality of patients’ care during the hospitalisation and, hence, surfaces as a new area of focus. This observational study evaluated the effectiveness of a pharmacist-led medication management via telephone in post-discharge patients to determine whether this intervention can be of use as a pragmatic mechanism to reduce the rates of 30-day hospital readmissions. With the aim of curbing healthcare expenditure through reducing 30-day readmission rates, the implementation of post-discharge interventions has emerged as a pivotal strategy to provide optimal health outcomes to patients in care transitions [14]. Multiple studies have evaluated various effective methods to ensure patients’ safety and reduce readmissions in post-discharge patients. With strategies to provide cost-effective interventions, a telephone follow-up (TFU) has been proposed by researchers as a relatively uncomplicated and plausible tool to maintain patient care without interrupting communication between healthcare providers and patients across continuity of care [15].

The findings of this study found that managing patients’ medicines after discharge through follow-up telephone calls were not associated with a reduction of 30-day hospital readmission. 11.3% of medical patients in the intervention group were readmitted to the hospital within 30-days post-discharge compared with 9% in the control group (*p* > 0.05). This current study had the similar conclusion to the following two studies. The previous studies assessed the impact of TFU on hospital readmission rates and reported that the TFU did not significantly benefit the rates of 30-day readmission. In an RCT study by *Dudas* et al. *(2001)*, 15% who received to the TFU was readmitted to the hospital within 30-days of discharge compared to 25% in the control group [2]. In another study by *Kilcup* et al. *(2013)*, 30-day readmission rate was 12% in the intervention group compared to 14% in the control group [14]. The previous studies showed a tendency towards readmissions without a statistical difference in patients who received the pharmacist TFU, which is dissimilar to the present study. The present study did not have the prospective control group to compare the readmission rates, which potentially have misled the results of the study. Additionally, the 30-day readmission rates in the current study showed lower than the previous studies. A direct relationship between the previous studies and the present study cannot be drawn because the previous studies were based in America where has the different health-care system, such as insurance or access to medicines, and may raise different system-associated problems in transitions of care. These factors, consequently, can contribute to various reasons and the number of hospital readmissions about the U.K. Another possibility not indicating the benefit of TFU in the current study could be that overall readmission rates were not high enough to show the differences. Amongst the reviewed studies showing a significant benefit of TFU, 30-day readmission rates for the control group ranged from the lowest 19.8*%* to the highest 38.1% [16, 18]. The overall readmission rates were 9% in the control group which could be relatively low at Buckinghamshire NHS Healthcare Trust. Readmissions were tracked only when patients were readmitted to Buckinghamshire Healthcare NHS Trust. Potential existed that patients could have been readmitted to other institutions, and it could mislead the rates of hospital readmissions.

In addition to the assessment of hospital readmission rates, this study provided insight into the prevalence of transitional-related medication problems as well as pharmacist engagement to manage any raised issues. With efforts to manage patients’ pharmaceutical problems, pharmacists made a wide variety of interventions through the TFU. A total of 192 patient-tailored interventions were made in 54 patients, equating to the average of 3.56 interventions per patient. The most common type of pharmacist interventions was the provision of patient counselling, made in 74% of patients, followed by non-adherence issues (in 53.7% patients), potential adverse drug reactions (in 50.0% patients). Others included instruction on duration, ensuring the supply of medicines, instruction on indications, advising dosage/directions, clarifying the medicines and reviewing potential drug interactions. Patient counselling was provided when patients asked questions during the intervention. This showed that many patients raised questions on their medicines after discharge. Pharmacists enabled to make early detection of the critical dispensing error (3 cases) through TFU, preventing patients from any critical harm. During the intervention, any uncertainties concerning their therapy regimens were referred to their physicians to discuss the issues. The implementation of the TFU built an opportunity for pharmacists to answer transitional-related or medicine-related questions as well as ensure patients’ safety.

The current study assessed the impact of interventions by consensus from two senior pharmacists. The assessment described the level of identified risks or incidents, multiplying estimates of consequences by the likelihood of the risk. 2.7% and 5.3% of the risk were categorised as catastrophic and major, respectively unless intervention was detected. 46.7% of the consequences were possible to recur. Then, the identified risk was scored according to the criteria by the NPSA Risk matrix. From risk scoring, moderate risk occurred most frequently (49.3%), translating into that the majority of pharmacist interventions were associated with solving moderate risk..Although it was unable to measure how many of the identified pharmaceutical problems could have been serious enough virtually to result in readmissions, or which degree of pharmacist interventions helped prevent patients from unplanned readmission, the risk assessment in the current study has important implication to provide prediction in this respect.

The time commitment involved for pharmacists to perform the TFU was an important contributing factor to ensuring the quality of the service. The mean duration of TFU was 5 to 20 min per patient interview. Pharmacists spent the more time on the first call (14.46 min. in the intervention group) than the 2nd call (8.07 min. in the intervention group). The more pharmaceutical problems identified the longer time was required for pharmacists to perform the TFU. The duration was dependent upon the number of pharmaceutical problems recognised by pharmacists during the TFU. For those who were not contacted, the time taken for the corresponding call was assumed to be one minute. The time input was justified for taking into account pharmacists’ time spent to prepare and make up to three attempts to reach patients. The current study measured the duration pharmacists spent solely for a patient interview. It excluded the time for pharmacists to complete documentation after the TFU interview or to make multiple attempts if patients were unavailable.

The second telephone call was of use to follow-up the issues raised by the first call and prevent further development of pharmaceutical problems. The current study looked at the occurrence of medication changes in patients’ therapy regimens between the first and second calls. Many patients had medicines changed following follow-up visit with their physician. Of 39 patients who had medication changes, 52% and 40% changes were ‘drug discontinued’ and ‘new drug started’, respectively. However, more than half the changes were related to the ‘discontinuation of drugs’. Reasons for the ‘discontinuation of drugs’ include the completion of drug (i.e., antibiotics, pain killer) and the therapeutic duplication of drug (i.e., same therapeutic class).

There were several limitations to the present study. It was not an RCT study in which design-associated limitations may be inherent. The current study has a small sample size in the intervention group. This study might be underpowered as only 62 patients were recruited due to limited time constraint. We were not able to compare baseline patient characteristics between the two groups as patient information in the control group was unavailable. There was a possibility of being different in patient characteristics between these two groups, which could have affected the rate of hospital readmission and misled the findings of the study. Moreover, hospital readmission rates were compared with the cohort discharged in the previous month. Seasonal variation may exist between two groups, potentially affecting the number of patients readmitted to the hospital. However, currently, there was no existing evidence demonstrating the different hospital readmission rates over 12 months to support such assumption. Although pharmacists have the framework of questions for the patient interview, there is still a possible risk that variability and inconsistency may exist for each patient in the process of data collection. Pharmacist interventions recommended to patients can differ depends on the individual pharmacist who makes telephone contacts. This study was only conducted in the two acute hospitals located in the UK, limiting the generalizability to other settings.

## Conclusion

The study concluded that the utility of pharmacist intervention with a focus TFU on managing patients’ medicines after discharge did not benefit in a reduction of 30-day readmissions. However, a pharmacist TFU intervention can be an effective tool to solve or avoid potential post-discharge pharmaceutical problems that patients encounter shortly after discharge. Pharmacists were able to educate and reinforce patients on the quality use of medicines through TFU. With their well-trained counselling skills and extensive medicine knowledge, the deployment of pharmacists can reap a benefit in managing patients’ medicines across the transitions of care. Therefore, a pharmacist TFU intervention could be proposed to promote optimal health outcome through patients’ medicines management support after hospitalisation. Future study needs to be focused on determining the target population (high-risk population) who would get the most benefit from a TFU intervention and the effectiveness of the intervention in the selected population.

## Abbreviations

n: Number
NHS: The National Health Service
RCT: Randomized controlled trial
SD: Standard deviation
TFU: Telephone follow-up
U.K.: The United Kingdom
Y: Years
